# Benign duodenojejunal junction stricture: a case report

**DOI:** 10.1097/MS9.0000000000002236

**Published:** 2024-06-04

**Authors:** Shishir Devkota, Niranjan Adhikari, Prajjwol Luitel, Sujan Paudel, Anil Suryabanshi, Santosh Dev, Abhishek Bhattarai

**Affiliations:** a Department of General Surgery, Tribhuvan University Teaching Hospital; b Maharajgunj Medical Campus, Institute of Medicine, Tribhuvan University, Kathmandu, Nepal

**Keywords:** case report, duodenojejunal junction, distal duodenal stricture, end-to-end anastomosis

## Abstract

**Introduction:**

Duodenojejunal stricture is a rare entity that has been attributed to peptic stricture, malignancy, chronic pancreatitis, Crohn’s disease and other benign causes.

**Case presentation:**

The authors present a case of a 67-year-old male who presented with upper abdominal pain for 2 weeks, 2 episodes of bilious vomiting, and inability to pass stool and flatus for 1 day. He had a history of chronic upper abdominal pain over the last 40 years and pulmonary tuberculosis 50 years back.

Computed tomography (CT) scan of the abdomen and pelvis showed short segment narrowing in the fourth segment of the duodenum with dilated first, second and third segment duodenal loops. Resection and end-to-end duodenojejunal anastomosis was performed and the outcome was normal.

**Discussion:**

Benign duodenojejunal can be treated with balloon dilatation, stenting, strictureplasty and resection anastomosis. Treatment should be offered considering efficacy, availability, complications of these modalities and aetiology.

**Conclusion:**

Anterograde push enteroscopy and CT scan can aid in preoperative diagnosis of duodenojejunal stricture. Even in older age groups without prior surgical history, benign duodenojejunal stricture can be the cause of intestinal obstruction. Resection and end-to-end duodenojejunal anastomosis can be safe and effective treatment modalities for duodenojejunal junction stricture.

## Introduction

Highlights

- Duodenojejunal junction is an uncommon site for stricture formation.
- Even in elderly age groups without prior surgical history, benign duodenojejunal stricture can be the cause of intestinal obstruction.
- Distal duodenal stricture can be a diagnostic dilemma; however, computed tomography (CT) scan of abdomen and anterograde push endoscopy can aid in preoperative diagnosis.

Distal duodenal obstruction (DDO) is clinically characterised by abdominal pain, bilious vomiting and radiologically by postbulbar obstruction^1^.

Causes of duodenal obstruction vary with patient’s age: duodenal atresia, webs, and annular pancreas being common in infancy, while peptic ulcer, Crohn’s disease, pancreatitis, superior mesenteric artery syndrome, and malignancy being more common in the adult population^2^. Duodenojejunal junction is an uncommon site for stricture formation, with causes being pancreatic carcinoma, duodenal adenoma, adenocarcinoma^3^, and benign stricture. Treatment options available for benign duodenojejunal stricture include stenting^4^, strictureplasty^5^, and resection anastomosis.

We present a case of intestinal obstruction due to duodenojejunal stricture managed with resection and anastomosis with normal outcome.

This case has been reported as per the 2023 SCARE guidelines^6^.

## Case presentation

A 67-year-old male, presented with colicky upper abdominal pain for 2 weeks, which was intermittent, aggravated by food, and relieved by analgesics. It was accompanied by 2 episodes of bilious vomiting and inability to pass stool and flatus for 1 day. He had burning type of upper abdominal pain intermittently over the last 40 years for which he used to take over-the-counter antacids and proton pump inhibitors. He had a history of pulmonary tuberculosis 50 years back, for which he completed a course of antitubercular medications. There was no history of significant weight loss, oral ulcer, diarrhoea, difficulty swallowing, perianal pain, blood in stool. He had no history of smoking, alcohol intake, diabetes mellitus, and family history of malignancy. He had no history of abdominal trauma, radiation exposure or abdominal surgery.

Clinical examination revealed a thin-built male with a body mass index of 17 kg/m^2^. His vitals were stable. Abdominal examination revealed soft, non-tender, non-distended abdomen, and rest systemic examination was unremarkable.

**Figure 1:**
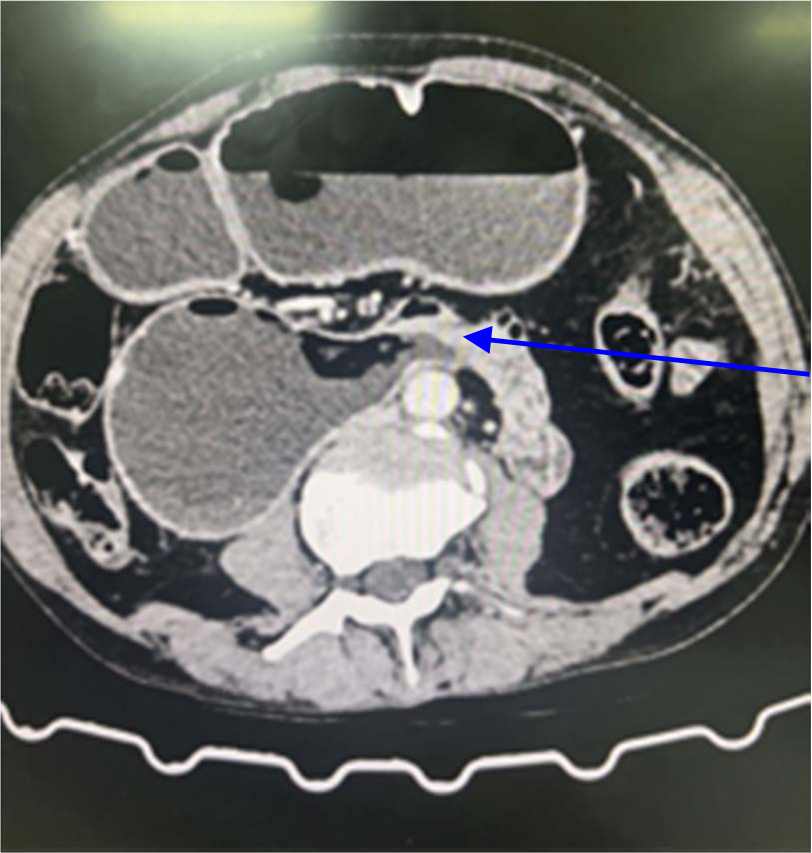
Computed tomography scan axial section showing homogenous, circumferential thickening at duodenojejunal flexure spanning 2.2 cm and thickness of 11 mm.

**Figure 2:**
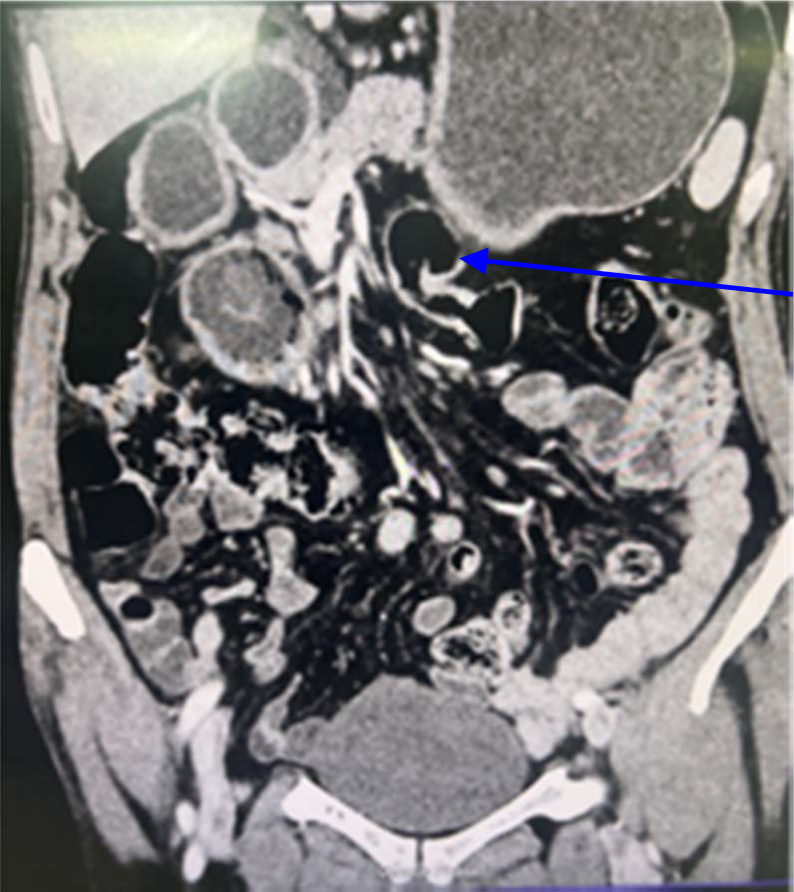
Computed tomography scan coronal section with arrow showing narrowing at duodenojejunal flexure.

On presentation, investigations showed haemoglobin: 15 g/dl, total leucocyte count: 8000/mm^3^, platelets: 307 000/mm^3^, serum sodium 137mEq/l, potassium 3.7mEq/l, urea 5 mmol/l, creatinine 58 μmol/l, Serum amylase 54U/l, serum lipase 21 U/l, serum albumin 34 gm/l, total bilirubin 12 μmol/l, direct bilirubin 2 μmol/l, aspartate aminotransferase 23 U/l, alanine aminotransferase 15 U/l. Ultrasonography showed a distended stomach with echogenic contents. Computed tomography (CT) scan of the abdomen and pelvis showed a short segment narrowing in the fourth segment of the duodenum without any obvious mass or enlarged lymph nodes. First, second and third segment duodenal loops were dilated Figure 1 and 2.

Anterograde push endoscopy showed narrowing in the fourth segment of the duodenum.

**Figure 3:**
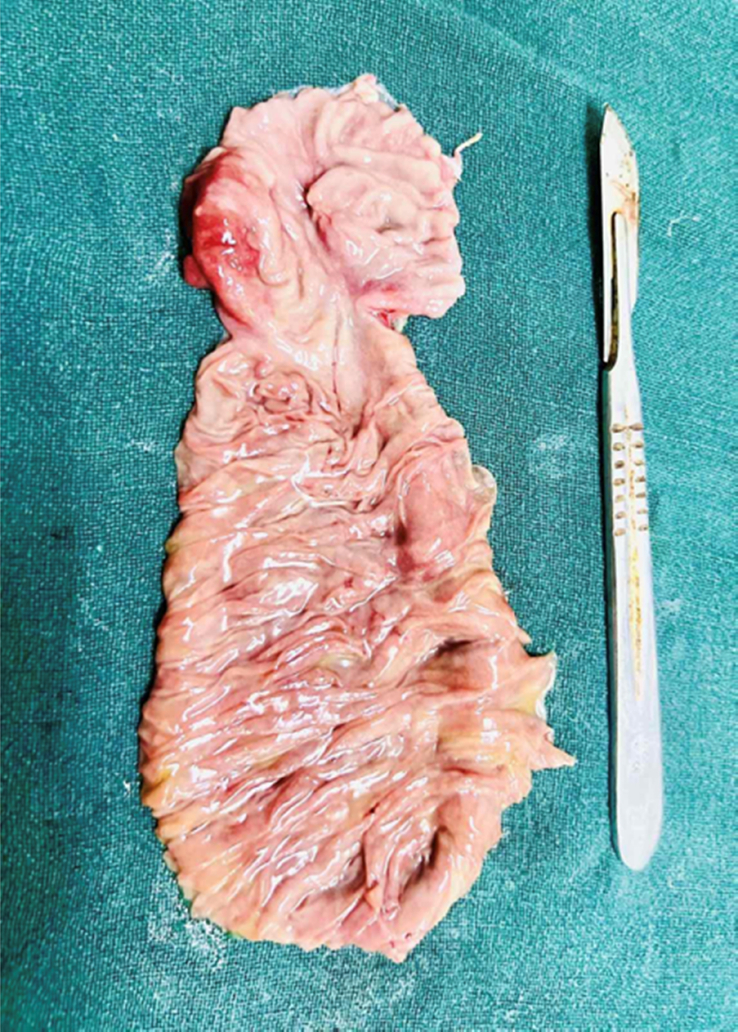
Circumferential thickening at duodenojejunal junction with significant luminal narrowing.

Resection with end-to-end duodenojejunostomy was performed under general anaesthesia. Intraoperative findings were distended stomach with dilated first, second and third segments of duodenum, circumferential thickening in duodenojejunal flexure, with significant luminal narrowing and no lymphadenopathy. Kocherisation was done to mobilise duodenum. Approximately 10 cm of proximal jejunum and 2.5 cm of the fourth part of the duodenum were resected (Figure 3).

**Figure 4:**
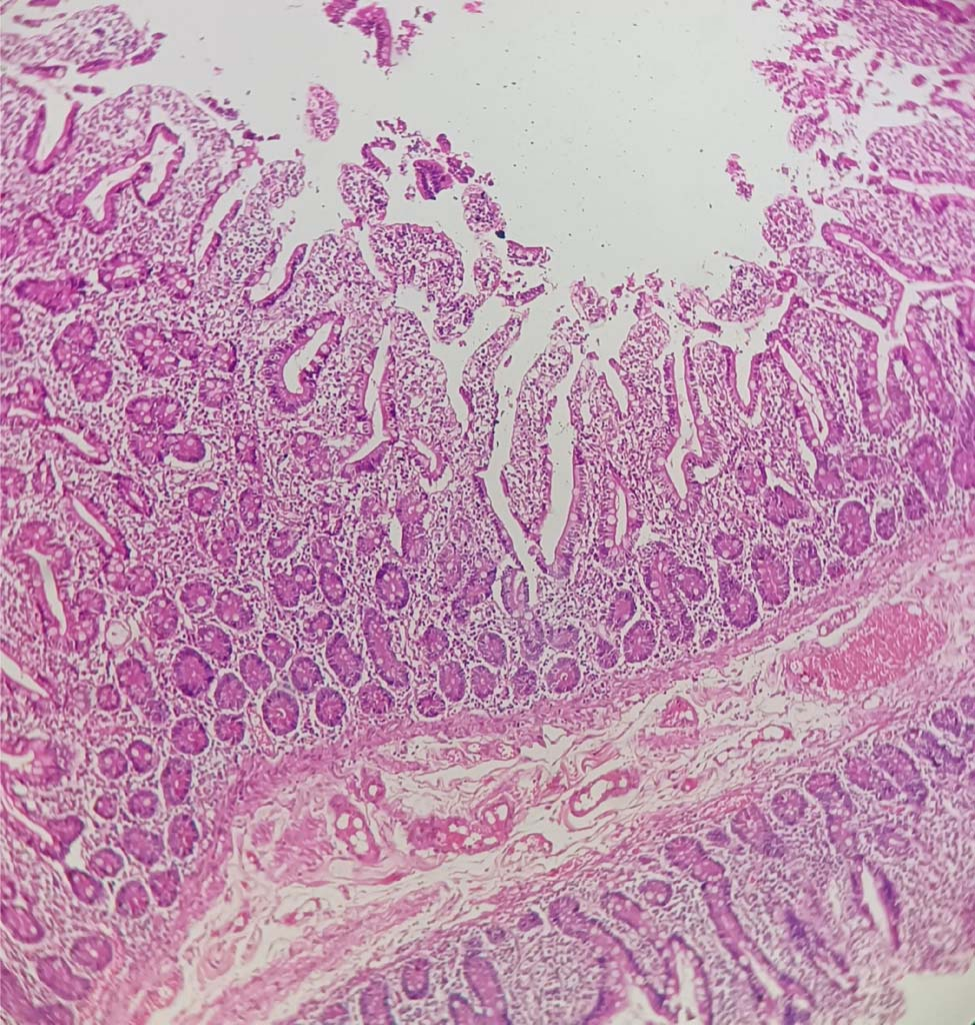
Sloughed off ciliated columnar lining epithelium of intestinal mucosa with lymphoplasmacytic and neutrophilic infiltrates in lamina propria.

Histopathology of the resected segments revealed ciliated columnar lining, mild lymphoplasmacytic infiltrates in lamina propria with plenty of neutrophils, without features of malignancy, granuloma and necrosis (Figure 4).

Witzel feeding jejunostomy was placed 40 cm distal to the anastomosis considering patient’s body mass index of 17 kg/m^2^, serum albumin level 34 gm/l and nutritional risk index of 89. Feeding was initiated on the 2nd postoperative day via feeding jejunostomy and per oral feeding from 8th postoperative day and patient was discharged subsequently. Postoperative stay was uneventful. Follow-up visits were done 2 weeks, 4 weeks, 6 weeks, 3 months and 6 months after the surgery. Feeding jejunostomy was removed after 6 weeks. Upper gastrointestinal endoscopy was found to be normal after 3 months and 6 months of surgery.

## Discussion

Duodenojejunal junction is an uncommon site for stricture formation as evidenced by its paucity of literature. Distal duodenal stricture has been attributed to multiple causes including peptic ulcer, chronic pancreatitis, Crohn’s disease, prior surgery, and pancreatic carcinoma. Tuberculosis has also been reported to be the cause of distal duodenal stricture^7^. However, none of the aforementioned causes could be identified in our case. Duodenojejunal stricture can be a diagnostic dilemma preoperatively, considering non-specific findings on ultrasonography and conventional upper gastrointestinal endoscopy; however, anterograde push endoscopy and computed tomography of the abdomen are useful diagnostic tools.

Non-malignant duodenojejunal stricture can be treated with balloon dilatation, stenting, strictureplasty, and resection anastomosis. Fluoroscopy-guided balloon dilatation is the least invasive modality among these having a shorter duration of hospital stay and efficacy of upto 71% but carries the risk of perforation and may require repeated dilatations^8^. Stenting of stricture using retrievable expandable stents has very limited evidence, especially regarding the timing of removal of stent, and has risks of restenosis and stent migration^4^. Strictureplasty is a less invasive surgical modality with fewer complications^9^ although upto 69% of patients may require reoperation^10^. Resection and end-to-end duodenojejunal anastomosis is a more invasive procedure with a longer duration of hospital stay and technical difficulties for mobilisation of intestines; however, it has a lower recurrence rate^11^. We opted for resection and anastomosis because of the possibility of malignancy and lack of expertise of other treatment modalities in our institution. Most of the studies conducted on the treatment of duodenojejunal stricture have small patient groups, hence larger studies need to be done to compare the efficacy of these treatment modalities.

## Conclusion

Distal duodenal stricture can be a diagnostic dilemma; however, anterograde push enteroscopy and CT scan can aid in preoperative diagnosis. Benign duodenojejunal junction stricture can be the cause of intestinal obstruction, even in older age groups without prior surgical history. Resection and end-to-end duodenojejunal anastomosis can be safe and effective treatment modalities for duodenojejunal junction stricture.

## Ethical approval

Since this is a case report, our Institutional Review Board waived the requirement for ethical approval.

## Consent

Written informed consent was taken from the patient for publication of this case report and accompanying images. A copy of the written consent is available for review by the Editor-in-chief of this journal on request.

## Source of funding

Not applicable.

## Author contribution

Sh.D., Sa.D. and A.B. are the treating surgeons. N.A. formulated the manuscript. P.L., S.P., and A.S. reviewed and edited the manuscript. All the authors reviewed and approved the final version of the manuscript.

## Conflicts of interest disclosure

All the authors declare that they have no conflict of interest.

## Research registration unique identifying number (UIN)

Not applicable.

## Guarantor

Shishir Devkota.

## Data availability statement

Not applicable.

## Provenance and peer review

Not applicable.

## Declaration

All the authors declare that the information provided here is accurate to the best of our knowledge.
